# Enhanced Zinc Intake Protects against Oxidative Stress and Its Consequences in the Brain: A Study in an In Vivo Rat Model of Cadmium Exposure

**DOI:** 10.3390/nu13020478

**Published:** 2021-01-31

**Authors:** Małgorzata M. Brzóska, Magdalena Kozłowska, Joanna Rogalska, Małgorzata Gałażyn-Sidorczuk, Alicja Roszczenko, Nazar M. Smereczański

**Affiliations:** Department of Toxicology, Medical University of Bialystok, 15-222 Bialystok, Poland; joanna.rogalska@umb.edu.pl (J.R.); malgorzata.galazyn-sidorczuk@umb.edu.pl (M.G.-S.); alicja.roszczenko@umb.edu.pl (A.R.); nazar.smereczanski@umb.edu.pl (N.M.S.)

**Keywords:** brain, cadmium, lipid peroxidation, oxidative/antioxidative balance, oxidative damage, oxidative stress, protection, protein oxidation, serum, zinc

## Abstract

We examined, in a rat model of moderate environmental human exposure to cadmium (Cd), whether the enhanced intake of zinc (Zn) may protect against Cd-caused destroying the oxidative/antioxidative balance and its consequences in the brain. The intoxication with Cd (5 mg/L, 6 months) weakened the enzymatic (superoxide dismutase, glutathione peroxidase, catalase) and non-enzymatic (total thiol groups, reduced glutathione) antioxidative barrier decreasing the total antioxidative status and increased the concentrations of pro-oxidants (hydrogen peroxide, myeloperoxidase) in this organ and its total oxidative status. These resulted in the development of oxidative stress and oxidative modifications of lipids and proteins. The co-administration of Zn (30 and 60 mg/L enhancing this element intake by 79% and 151%, respectively) importantly protected against Cd accumulation in the brain tissue and this xenobiotic-induced development of oxidative stress and oxidative damage to lipids and proteins. Moreover, this bioelement also prevented Cd-mediated oxidative stress evaluated in the serum. The favorable effect of Zn was caused by its independent action and interaction with Cd. Concluding, the enhancement of Zn intake under oral exposure to Cd may prevent the oxidative/antioxidative imbalance and oxidative stress in the brain and thus protect against injury of cellular macromolecules in the nervous system.

## 1. Introduction

Zinc (Zn) is a bioelement playing a very important role in living systems [1,2,3,4]. This micronutrient is responsible for the proper activity of numerous enzymes (including antioxidative enzymes), growth, differentiation, and metabolism of cells, synthesis of protein and deoxyribonucleic acid (DNA), immunity, wound healing, and many other functions [2,3,4,5]. Supplementation with Zn is recommended in the management of a great number of health conditions, such as acrodermatitis enteropathica, Wilson’s disease, common cold, acne, diarrhea, epilepsy, and to support the immune system [1,4,6,7]. Although Zn is present in a wide variety of food items, it has been estimated that deficiency of this essential micronutrient may affect even up to 20% of the World’s population [8]. Because Zn undernutrition in humans may result in very serious health consequences [1,4,5,7,9], the consumption of food products rich in this bioelement is widely recommended by nutritionists and health promoters [1,4]. However, it is also important to notice that in the available literature, there are reports on negative health outcomes of excessive supplementation with this element, such as disturbances in the homeostasis of other bioelements, especially copper (Cu), leading to Cu-deficiency anemia, decrease in the activity of Cu-dependent enzymes, and changes in the metabolism of cholesterol [4,6]. Zn plays a critical role in neurodevelopment and both its deficiency and excess are neurotoxic and are involved in the pathogenesis of neurological diseases such as amyotrophic lateral sclerosis, depression, schizophrenia, Parkinson’s disease, and Alzheimer’s disease [5,7].

Available data, including the findings of our studies, indicate many beneficial effects of Zn administration also in the alleviation of toxicity of heavy metals, including cadmium (Cd) [10,11,12,13,14,15,16,17,18,19,20,21,22]. Chronic exposure to Cd of the general population is a global health concern and, despite numerous efforts to restrict its release into the environment, prognoses suggest that human’s intoxication with this toxic element will elevate because it does not undergo biodegradation [23]. Food is the main source of environmental exposure to Cd of tobacco non-smokers [23,24]. The unfavorable influence of environmental intoxication with this xenobiotic manifests first of all in the kidney and skeleton damage [23,25]. However, epidemiological studies also show a link between the exposure and liver injury [23,26], cardiovascular disorders [23,27], cancer of the lung, breast, pancreas, and prostate [25,28], as well as damage to the brain, including neurodegenerative illnesses such as Parkinson’s and Alzheimer’s diseases [29,30,31].

Recently growing attention has been focused on Cd as a risk factor for neurodegenerative diseases [30,31,32,33]. Although so far the unfavorable influence of Cd on the nervous system has been revealed in only a few epidemiological studies [29,30,31], this effect has been reported in some investigations carried out in animal models [18,22,31,34,35,36,37,38,39,40,41,42,43,44,45,46]. The injurious action of this heavy metal on the central nervous system is multidirectional and complex [18,22,31,34,35,36,37,38,39,40,41,42,43,44,45,46,47,48]. Literature data indicate that Cd may stimulate the generation of reactive oxygen species (ROS) leading to oxidative stress and interact with essential elements (including calcium—Ca) in the nervous tissue, as well as impair neurogenesis, induce apoptosis of neurons, and influence the glutamatergic system, gene expression, and homeostasis of neurotransmitters, including acetylcholinesterase [18,22,31,34,35,36,37,38,39,40,41,42,43,44,45,46,47,48]. It is known that lipids are among cellular macromolecules most vulnerable to oxidative modifications [38,39,49] and therefore, the brain tissue, due to the abundance of lipids and high oxygen utilization, is especially susceptible to damage by pro-oxidants via the oxidative stress-related mechanisms [22,38,39,50].

Numerous investigations have shown that Zn can decrease Cd content in the body [10,11,12,17] and alleviate its injurious action in various organs and systems, mainly the kidney, liver, and bone tissue [10,11,12,13,14,15,16,17,20,21]. The mechanism of Zn-mediated protection towards the harmful action of Cd has not been completely explored yet. However, the involvement in these pathways of direct competition between these two metals for the systems of transport and sites of binding in cells, Zn-induced synthesis of metallothionein (MT), and strong antioxidative potential of this bioelement is well established [11,12,14,15,16,17,18,20,21,22,37]. Zn, as a component of the active center of an antioxidative enzyme—superoxide dismutase (SOD)—and a factor decreasing the activity of oxidases, plays a very important role in the maintenance of the balance between pro- and antioxidants in cells [2,3,17]. Moreover, this element may also up-regulate the activation of antioxidant transcription factors and the expression of genes for antioxidants [2,3,17,20].

In our previous investigations carried out in the rat model of moderate and relatively high chronic human intoxication with Cd (5 and 50 mg/L, respectively, for 6 and 12 months) we have noted that the enhancement of the intake of Zn (30 and 60 mg/L) lowered Cd accumulation in the body (Table S1) and prevented this xenobiotic-induced damage to the bone tissue and liver, including the action resulting from its pro-oxidative properties [10,11,12,13,14,15]. Taking into account strong antioxidative ability of Zn [2,3], as well as the results of our previous studies [10,11,12,13,14,15,16] and available literature data [17,18,19,20,21,22] showing the protective effects of this bioelement against Cd toxicity, we have hypothesized that the increased consumption of Zn under the repeated exposure to Cd may also prevent oxidative stress and its consequences in the nervous tissue. Results of some authors suggest that Zn may alleviate the pro-oxidative action of Cd in the brain [18,22]. However, the data come from studies carried out in experimental models that are not in line with the general population exposure to this xenobiotic and do not involve a complex estimation of the balance between pro- and antioxidants. To scrutinize our hypothesis, in the present study numerous markers of the enzymatic (SOD, glutathione peroxidase—GPx, glutathione reductase—GR, and catalase—CAT) and non-enzymatic (total thiol groups—TSH, protein thiol groups—PSH, and reduced glutathione—GSH) antioxidative barrier, total antioxidative status (TAS), and indices of oxidative status (hydrogen peroxide—H_2_O_2_, myeloperoxidase—MPO, total oxidative status—TOS, and oxidative stress index—OSI) of the brain tissue, as well as the biomarkers of oxidative damage to the cellular macromolecules, such as lipids (lipid peroxides—LPO, malondialdehyde—MDA, and 8-isoprostane) and proteins (protein carbonyls—PC), were evaluated in the brain tissue in the previously used in our research rat model of repeated moderate human intoxication with Cd [10,11,12,13,14,15]. The wide range of measured parameters allowed the estimation of the effect of the treatment with this heavy metal on the oxidative/antioxidative status of the brain and the possible protective impact of Zn towards this xenobiotic-caused imbalance between anti- and pro-oxidants in the nervous tissue. Taking into consideration that excess of Zn in the organism may be dangerous for health, including the nervous system [1,3,4,5,6], the important aim of the current investigation was also the estimation of the influence of this bioelement administration without co-treatment with Cd on the brain oxidative/reductive status. TAS, TOS, and the severity of oxidative stress (estimated on the base of the value of OSI) were assessed not only in the brain but also in the serum to find whether there exists a connection between the oxidative/antioxidative status in these two compartments of the body. Because it is impossible to evaluate the redox status of the brain of a living individual without some invasive procedures, revealing a dependence between the redox status of the brain and serum would be very useful in forecasting the oxidative/antioxidative imbalance in the nervous system. To explain the possible pathways of the independent and interactive impact of Zn and Cd, both metals concentrations in the brain tissue were assayed as well.

To the best of our knowledge, a similar study has not been carried out and it was expected to bring new relevant data on the possibility of the use of Zn supplementation in the protection against Cd-caused imbalance between pro- and antioxidants in the brain and its consequences. Because oxidative stress is the mechanism of neurotoxic action of Cd [22,31,35,37,38,39,40,41,42,43,44,45,47,48], revealing that enhanced intake of Zn protects from destroying the oxidative/antioxidative status of the brain could indicate that this bioelement may ameliorate the injurious influence of this toxic heavy metal on the nervous system.

## 2. Materials and Methods

### 2.1. Animals

The experiment was approved by the Local Ethics Committee for Animal Experiments in Bialystok (approval No 9/2007 issued on February 28, 2007). All procedures involving animals were performed following the ethical principles and institutional guidelines, as well as the International Guide for the Use of Animals in Biomedical Research.

The study was performed on 48 adult (10 weeks old) male Wistar rats (Crl: WI (Han); certified Laboratory Animal House in Brwinów, Poland) housed in controlled conventional conditions (temperature 22 ± 2 °C, humidity 50 ± 10%, 12/12 h light/dark cycle). The animals had free access to the LSM dry diet intended for rodents (Agropol, Motycz, Poland), containing 48 μg Zn/g (according to the producer) and 0.098 μg Cd/g (own data [10]) and drinking water free of contaminants (≤0.05 μg Cd/L and ≤10 μg Zn/L [10]).

### 2.2. Sources of Cd Exposure and Zn Supplementation

The animals were administered with Cd and Zn in drinking water. Cd was given as cadmium chloride (CdCl_2_ × 2½ H_2_O; POCh, Gliwice, Poland) at the concentration of 5 mg Cd/L, whereas Zn was supplemented as zinc chloride (ZnCl_2_; Merck, Darmstadt, Germany) at the concentration of 30 and 60 mg Zn/L. The aqueous solutions of Cd or/and Zn were prepared by dissolving appropriate amounts of CdCl_2_ and ZnCl_2_ in redistilled water to make solutions of 1000 mg Cd/L and 1000 mg Zn/L used for the preparation of solutions containing Cd or/and Zn at the needed concentrations.

### 2.3. Study Protocol

The rats were assigned (randomly) into the following six groups (each composed of 8 animals):

- Control: Received drinking water without the addition of Cd and Zn and the standard rodent chow;
- Zn 30: Received drinking water containing 30 mg Zn/L;
- Zn 60: Received drinking water containing 60 mg Zn/L;
- Cd: Treated with Cd in drinking water at the concentration of 5 mg/L;
- Cd + Zn 30: Received drinking water containing Cd and Zn at the concentrations of 5 mg/L and 30 mg/L, respectively; and
- Cd + Zn 60: Received drinking water containing Cd and Zn at the concentrations of 5 mg/L and 60 mg/L, respectively.

The experiment lasted 6 months.

**Figure 1:**
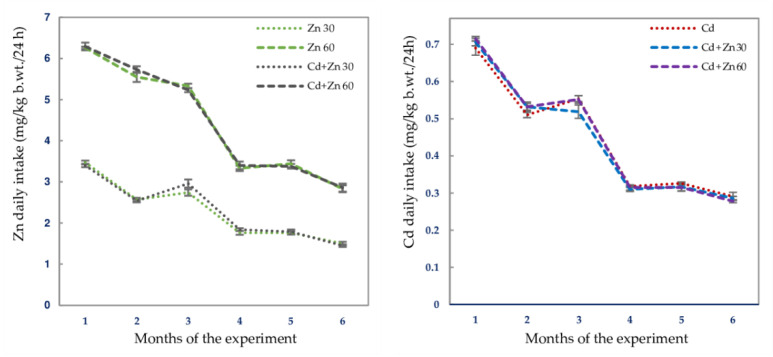
The mean daily intakes of zinc (Zn) and cadmium (Cd) in the subsequent months of their 6-month administration at the concentrations of 30 or 60 mg Zn/L and 5 mg Cd/L alone and together. Data represent mean ± standard error (SE) for 8 animals.

Cd intake in particular experimental groups that received this heavy metal via drinking water alone and together with Zn (Cd group, Cd + Zn 30 group, and Cd + Zn 60 group) did not differ (Table 1, Figure 1). The mean Cd intake by rats intoxicated with this xenobiotic with or without simultaneous supplementation with Zn during the experiment reached 0.430 ± 0.019 mg/kg body weight (b.w.) (mean ± standard error (SE)) per day. The intoxication with 5 mg Cd/L reflects moderate environmental human exposure, which was confirmed by this xenobiotic concentration in the blood (1.001–2.064 μg Cd/L) and urine (0.0064–0.0197 μg Cd/24 h) of the rats [10] being within the range of values detected in the general population [23,25,26,27,28,30].

The level of Zn administration was selected to provide its enhanced, but not too high, intake. The mean daily Zn intake during the whole study period was 2.220 ± 0.084 mg/kg b.w. (mean ± SE) in the animals administered with 30 mg Zn/L of drinking water and 4.475 ± 0.407 mg/kg b.w. in the ones supplemented with 60 mg Zn/L alone or together with the intoxication with Cd and it was the same irrespective of whether the two elements were given separately or jointly (Figure 1, Table 1) [10]. As it was estimated based on Zn concentration in drinking water and the LSM diet and their consumption monitored throughout the study, the administration of 30 and 60 mg Zn/L allowed to increase this micronutrient daily intake by 79% and 151%, respectively, compared to its amount provided via the standard LSM chow (1.186 ± 0.019 mg/rat; mean ± SE).

The consumption of the LSM chow was within the same range in the rats of the six groups and therefore Cd and Zn intakes via food were comparable in all animals.

At termination, all animals from each group (there were no deaths throughout the 6-month experiment) were sectioned under intraperitoneal (i.p.) anesthesia by the use of Vetbutal at the dose of 30 mg/kg b.w. (pentobarbital sodium and pentobarbital 5:1; Biowet, Pulawy, Poland). The whole blood was taken by the heart puncture (with or without heparin as an anticoagulant; Biochemie, GmbH, Kundl, Austria), and the brain, as well as other organs, were collected. The whole blood taken without heparin, after coagulation, was subjected to centrifugation (MPW-350R centrifugator, Medical Instruments, Warsaw, Poland) and then serum was separated. The biological material, including the brain and serum used in the present investigation, was stored frozen (−70 °C).

The used rat model has been described in more detail in our previous reports [10,11,12,13,14,15].

### 2.4. Estimation of the Oxidative/Antioxidative Status of the Brain and Serum

#### 2.4.1. Preparation of the Brain Tissue Homogenates

Homogenates of the brain (10%; weight/volume) were prepared by blending the sections of this organ (of known weight) with an ice-cold potassium phosphate buffer (50 mM, pH = 7.4; made up with the use of dipotassium hydrogen phosphate, potassium dihydrogen phosphate [POCh], and distilled water) and butyl-hydroxytoluene (Sigma-Aldrich GmbH, Steinheim, Germany) in a glass/teflon homogenizer (Schütt homogen^plus^, Schutt Labortechnik GmbH, Göttingen, Germany). Homogenates prepared for the assay of SOD, GPx, and GR were subjected to centrifugation (MPW-350R centrifugator, Medical Instruments, Warsaw, Poland) at 20000× *g* for 30 min (4 °C), whereas other measurements were performed in the aliquots of the homogenates centrifuged for 20 min at 700× *g* (4 °C) [49]. The received aliquots of the brain tissue were stored frozen (−70 °C) until the assay.

#### 2.4.2. Assay of the Markers of the Oxidative/Antioxidative Status and Quantification of the Severity of Oxidative Stress in the Brain

All measurements with the use of commercial kits were carried out according to the recommendations of producers. The precision of the measurements was expressed as the intra-assay coefficient of variation (CV). The spectrophotometer MULTISCAN GO (Thermo Scientific, Vantaa, Finland) was used in the quantification of the variables determined. All investigated parameters were adjusted for protein concentration quantified using the BioMaxima kit (Lublin, Poland) with the CV < 3%.

##### Antioxidants in the Brain

The activity of SOD in the brain was assayed with the use of the kit no. 706002 by Cayman Chemical Company (Ann Arbor, MI, USA) which utilizes a tetrazolium salt to detect superoxide radicals (O_2_^−^) that are generated by hypoxanthine and xanthine oxidase (CV < 3.5%). CAT was determined spectrophotometrically as described by Aebi [51] (CV < 4.5%). The activities of glutathione-related enzymes such as GPx and GR were determined using the Bioxytech GPx-340 (Catalog no. 21017) and GR-340 (Catalog no. 21018) kits by OxisResearch (Burlingame, CA, USA) (CV < 4% and <2.5%, respectively). The kit no. 21017 measures the rate of GPx-induced recycling of oxidized glutathione (GSSG) to its reduced form (GSH). The base of the assay with the use of the kit no. 21018 is the reaction of reduced nicotinamide adenine dinucleotide phosphate (NADPH) oxidation to NADP^+^, which is catalyzed by GR.

The Glutathione Assay Kit no. 703002 by Cayman Chemical Company (Ann Arbor, MI, USA) was used for colorimetrical measurement of GSH and GSSG concentrations (CV < 2.3% and <3.5%, respectively). GSH assay is based on the reaction of GSH and 5,5′-dithio-bis-2-nitrobenzoic acid and the measurement (at 405–412 nm) of the absorbance of its product—5-thio-2-nitrobenzoic acid. The concentration of GSSG was evaluated by derivatization of its reduced form (GSH) performed with 2-vinylpyridine. The ratio of the concentrations of reduced and oxidized forms of glutathione (GSH/GSSG) was counted.

The concentrations of PSH and TSH were estimated following the method by Ellman [52] (CV < 3% and <3.5%, respectively).

The assay of TAS was performed with the ImAnOx (TAS) enzyme-linked immunosorbent assay (ELISA) kit no. KC5200 by Immundiagnostik AG (Bensheim, Germany) (CV < 1.5%). The quantification is based on a reaction between a defined amount of H_2_O_2_ added into an investigated sample and antioxidants present in it. The residual H_2_O_2_ forms products absorbing at 450 nm. The certified TAS values in the samples included in the kit used for this parameter assay in the brain were 208–282 and 254–344 μmol/L, while the values quantified by us reached 226 ± 2.94 and 307 ± 3.71 μmol/L (mean ± standard deviation (SD)), respectively.

##### Pro-Oxidants in the Brain

The concentration of H_2_O_2_ in the brain was quantified with the Bioxytech H_2_O_2_-560 kit no. 21024 by OxisResearch (Portland, OR, USA) (CV < 4%). The assay is based on the reaction of oxidation of divalent iron (Fe) to its trivalent form. MPO was determined by the Rat (MPO) double-antibody sandwich ELISA kit no. 201-11-0575 by SunRed (Shanghai, China) (CV < 3%).

The PerOx (TOS) ELISA kit no. KC5100 by Immundiagnostik AG was used to the assay of the value of TOS based on the measurement (at 450 nm), in the reaction with peroxidase, of total LPO (CV < 2.5%). The values of TOS in the control samples were 164–304 and 292–488 μmol/L, while the values determined by us reached 234 ± 4.86 and 406 ± 9.85 μmol/L (mean ± SD), respectively. Oxidative stress index was mathematically estimated as the ratio of the values of TOS and TAS (OSI = TOS/TAS).

##### Indices of Oxidative Injury of Lipids and Proteins

The determinations of the concentrations of LPO and 8-isoprostane were made with the Bioxytech LPO-586 kit no. 21012 provided by OxisResearch (Burlingame, CA, USA) (CV < 6.5%) and 8-isoprostane Enzyme Immunoassay (EIA) kit no. 516351 by Cayman Chemical (CV < 6%), respectively. The quantification of LPO consists in the measurement, at 586 nm, of the absorbance of the product of the reaction of MDA and 4-hydroxyalkenal, present in the investigated sample, with added N-methyl-2-phenylindole. The concentration of MDA was assayed by a spectrophotometric method by Buege and Aust [53] (CV < 3%), based on its reaction with thiobarbituric acid in which a pink complex with a maximum of absorption at 535 nm is formed.

The determination of PC concentration was performed spectrophotometrically in the reaction of PC, present in the investigated sample, with added 2,4-dinitrophenylhydrazine [54] (CV < 3.5%).

#### 2.4.3. Determination of the Severity of Oxidative Stress in the Serum

The serum TAS was measured using the ImAnOx (TAS) ELISA kit (Catalog no. KC5200) by Immundiagnostik AG (Bensheim, Germany) (CV < 2.5%). The certified TAS values in the controls included in the kit were 208–282 and 254–344 μmol/L, while the values quantified by us reached 221 ± 5.46 and 301 ± 7.22 μmol/L (mean ± SD), respectively. TOS was assayed by the PerOx (TOS) ELISA kit (Catalog no. KC5100) by Immundiagnostik AG (CV < 2.5%). The certified values of TOS were 108–200 and 305–509 μmol/L, and the values determined by us reached 163.2 ± 2.97 and 414.9 ± 8.54 μmol/L (mean ± SD), respectively. Like in the brain tissue, the serum OSI was evaluated as the ratio of TOS/TAS.

### 2.5. Determination of the Concentration of Zn and Cd in the Brain

Slices (0.15–0.20 g) of the brain tissue (always collected from this same area of the brain), after weighing (accuracy to 0.0001 g), were submitted to wet digestion with suprapure concentrated nitric acid (65% HNO_3_; Merck, Darmstadt, Germany) and chloric acid (35% HCl; Merck), at the ratio of 9:1, performed with the use of a microwave system (Multiwave, Anton Paar GmbH, Graz, Austria) [10]. After the microwave digestion, the excess of acids were removed from the samples by evaporating under their slight warming up. Afterward, the samples were diluted with ultra-pure water (taken from the water purification MAXIMA system; ELGA, Bucks, UK).

The concentrations of Zn and Cd in the samples of the brain tissue, prepared as above described, were determined (after appropriate dilution if it was needed) by the atomic absorption spectrophotometry method (AAS method). An atomic absorption spectrophotometer model Z-5000 by Hitachi (Tokyo, Japan) with Zn and Cd cathode lamps (resonance lines: 213.8 nm for Zn and 228.8 nm for Cd) by Photron (Narre Warren, Australia) was used. The concentration of Zn was assayed by the flame AAS (F AAS; atomization in an air-acetylene burner), whereas Cd was measured by the flameless AAS with electrothermal atomization in a graphite furnace (GF AAS). The limit of detection of Zn was 10 μg/L, while that of Cd was 0.02 μg/L. The brain concentrations of both elements are given in μg per gram of wet tissue weight.

The analytical quality of the assay of the concentrations of Zn and Cd was monitored by assaying these elements in the Standard Reference Material no. 1577b—Bovine Liver (National Institute of Standards and Technology, Gaithersburg, MD, USA). Both metals concentrations determined in our laboratory in the certified material (131 ± 10 μg Zn/g and 0.484 ± 0.032 μg Cd/g; mean ± SD) agreed with the reference values (127 ± 16 μg Zn/g and 0.50 ± 0.03 μg Cd/g). The recovery for Zn and Cd was 103% and 97%, respectively, whereas the CV was <7.6% and <6.6%, respectively.

### 2.6. Statistical Analysis

Statistical elaboration of the received data was carried out with the Statistica 12 package (StatSoft, Tulsa, USA). The Kruskal-Wallis signed-rank nonparametric test was done to disclose if there were any differences among the investigated six groups (*p* < 0.05) and then the Kruskal-Wallis post hoc test was performed to reveal statistically significant (*p* < 0.05) differences between every two means. Statistically significant differences of the investigated parameters vs. the control, the group administered only with Cd (Cd + Zn 30 group vs. Cd group and Cd + Zn 60 group vs. Cd group), the group administered only with Zn (Cd group vs. Zn 30 group, Cd + Zn 30 group vs. Zn 30 group, Cd group vs. Zn 60 group, and Cd + Zn 60 group vs. Zn 60 group), and the respective group administered with 30 mg Zn/L of drinking water separately or together with Cd (Zn 60 group vs. Zn 30 group and Cd + Zn 60 group vs. Cd + Zn 30 group) are presented in figures. To discern the occurrence of the interactive action of both elements at their co-treatment (Cd + Zn 30 group and Cd + Zn 60 group), a two-way analysis of variance (ANOVA/MANOVA) was carried out and if such interaction was noted, its potential character was estimated as well. The nature of Cd-Zn interaction (additive, antagonistic, or synergistic [55]) was evaluated on the base of comparison between the effects of simultaneous administration of these elements (Cd + Zn 30 group and Cd + Zn 60 group) and the value of the effects occurring when they were administered alone (Cd group, Zn 30 group, and Zn 60 group). Spearman rank correlation was applied to recognize the relationships between two various parameters. A correlation is considered statistically significant in the case when a correlation coefficient (*r*) has *p* < 0.05.

## 3. Results

### 3.1. The Impact of Cd and/or Zn on the Enzymatic Antioxidative Status of the Brain

**Figure 2:**
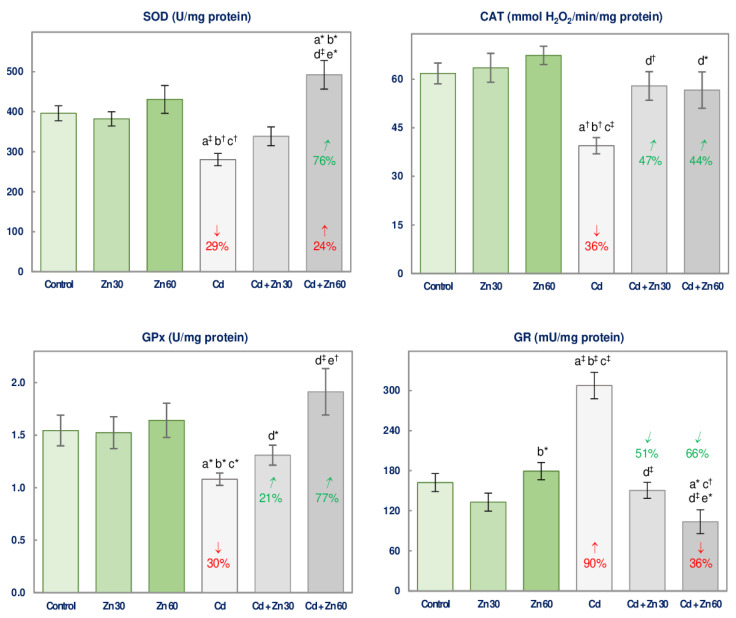
The activities of superoxide dismutase (SOD), glutathione peroxidase (GPx), glutathione reductase (GR), and catalase (CAT) in the brain tissue of male rats after 6-month treatment with cadmium (5 mg Cd/L) and/or zinc (30 or 60 mg Zn/L) via drinking water. Data are shown as mean ± standard error (SE) for 8 animals. Statistically significant differences: a vs. Control group; b vs. Zn 30 group; c vs. Zn 60 group; d vs. Cd group; e vs. Cd + Zn 30 group; * p < 0.05; ^†^ *p* < 0.01; ^‡^ *p* < 0.001. The values inside the bars (or above them) express the percentage changes vs. the control (↓, decrease; ↑, increase;) or Cd group (↙, decrease; ↗, increase).

The 6-month supplementation with Zn at both concentrations (30 and 60 mg/L of drinking water) alone did not influence the activities of assayed antioxidative enzymes (SOD, GPx, GR, and CAT) in the brain (Figure 2).

The administration of the drinking water containing 5 mg Cd/L for 6 months led to a decline (by 29–36%) in the activities of SOD, GPx, and CAT and an elevation (by 90%) in GR activity compared to the control rats (Figure 2).

The co-administration of 30 or 60 mg Zn/L to the animals treated with Cd entirely counteracted this xenobiotic-induced changes in the activities of all assayed antioxidative enzymes (Figure 2). Moreover, the co-treatment with Zn at the higher concentration made the activities of SOD and GR higher (by 24%) and lower (by 36%), respectively, compared to the control (Figure 2). The activities of SOD and GPx were higher, while GR activity was lower in the Cd + Zn 60 group than in the Cd + Zn 30 group (Figure 2). According to the ANOVA/MANOVA analysis the impact of Zn administration on the brain activity of antioxidative enzymes under the intoxication with 5 mg Cd/L resulted from the main effect (independent influence) of Zn (*F* = 7.307–35.80, *p* < 0.05–0.001) and/or its interactive action with Cd (*F* = 5.000–43.30, *p* < 0.05–0.001), which was antagonistic (Table 2). The two-way analysis of variance revealed no independent impact of 30 mg Zn/L and/or its interaction with Cd on SOD and GPx activities (Table 2) despite the favorable effect of this bioelement supplementation noted based on the Kruskal-Wallis test (Figure 2).

### 3.2. The Impact of Cd and/or Zn on the Non-Enzymatic Antioxidative Status of the Brain

**Figure 3:**
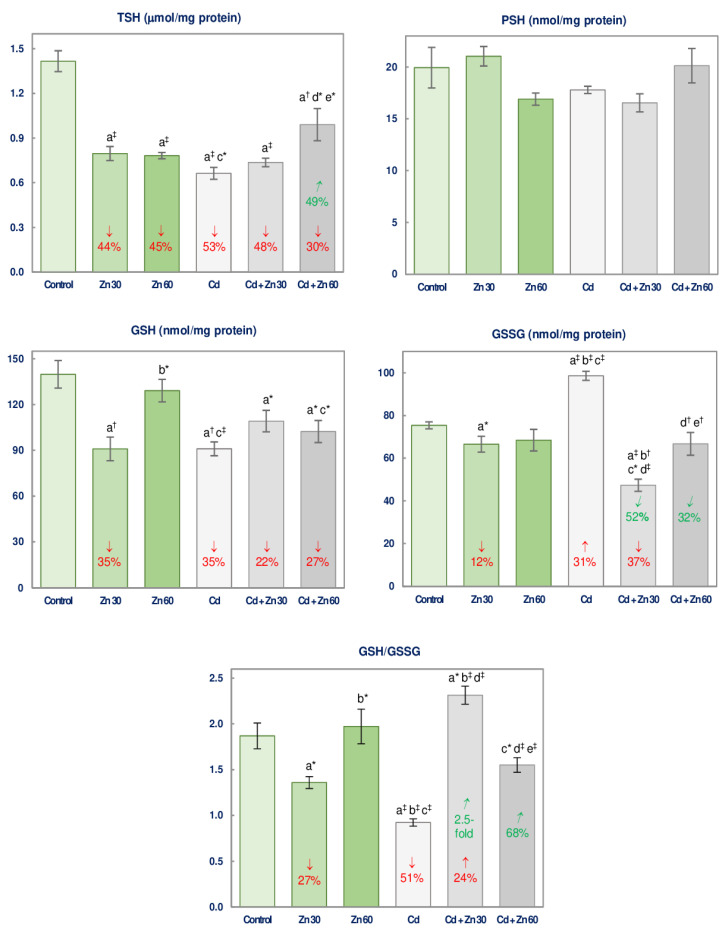
The concentrations of total thiol groups (TSH), protein thiol groups (PSH), reduced glutathione (GSH), and oxidized glutathione (GSSG), as well as the GSH/GSSG ratio in the brain tissue of male rats after 6-month treatment with cadmium (5 mg Cd/L) and/or zinc (30 or 60 mg Zn/L) via drinking water. Data are shown as mean ± standard error (SE) for 8 animals. Statistically significant differences: a vs. Control group; b vs. Zn 30 group; c vs. Zn 60 group; d vs. Cd group; e vs. Cd + Zn 30 group; * p < 0.05; ^†^ *p* < 0.01; ^‡^ *p* < 0.001. The values inside the bars express the percentage changes or fold of changes vs. the control group (↓, decrease; ↑, increase;) or Cd group (↙, decrease; ↗, increase).

The administration of 30 mg Zn/L caused a decrease (by 12–44%) in the brain concentrations of TSH, GSH, and GSSG, and GSH/GSSG ratio, while the higher dosage of Zn (60 mg/L) resulted only in a decline (by 45%) in TSH concentration (Figure 3).

The treatment with Cd (5 mg/L) alone led to a reduction (by 35–53%) in the concentrations of TSH and GSH and the GSH/GSSG ratio, as well as to an enhancement (by 31%) of GSSG concentration (Figure 3).

The application of Zn at the concentration of 30 mg/L during the treatment with 5 mg Cd/L provided complete protection against the impact of this heavy metal on GSSG concentration and GSH/GSSG ratio making them even lower (by 37%) and higher (by 24%), respectively, than in the control group (Figure 3). However, it did not influence the concentrations of TSH and GSH (Figure 3). The higher Zn supplementation completely or partially prevented the Cd-mediated alterations in TSH and GSSG concentrations, and the ratio of GSH/GSSG (Figure 3). The concentrations of TSH and GSSG were higher and the GSH/GSSG ratio was lower in the Cd + Zn 60 group than in the Cd + Zn 30 group (Figure 3). The ANOVA/MANOVA analysis revealed that the influence of Zn on the levels of non-enzymatic antioxidants in the brain resulted from the independent impact of this essential element (*F* = 4.468–31.31, *p* < 0.05–0.001) and its interactive action with Cd (*F* = 4.364–102.3, *p* < 0.05–0.001) (Table 3). The analysis showed that the decline in GSH concentration in the Cd + Zn 60 group was a consequence of the independent Cd action (*F* = 27.35, *p* < 0.001; Table 3).

PSH concentration was unaffected in all experimental groups administered with Cd and/or Zn (Figure 3).

### 3.3. The Impact of Cd and/or Zn on Pro-oxidants Concentration in the Brain

**Figure 4:**
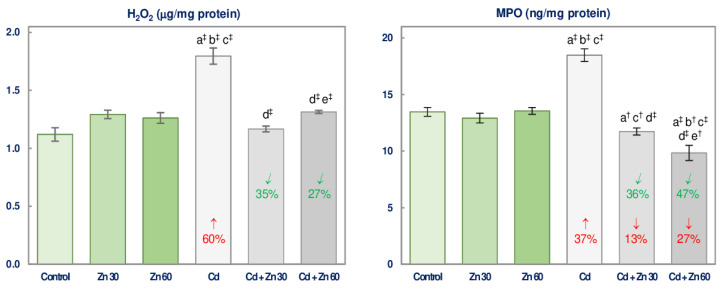
The concentrations of hydrogen peroxide (H_2_O_2_) and myeloperoxidase (MPO) in the brain tissue of male rats after 6-month treatment with cadmium (5 mg Cd/L) and/or zinc (30 or 60 mg Zn/L) via drinking water. Data are shown as mean ± standard error (SE) for 8 animals. Statistically significant differences: a vs. Control group; b vs. Zn 30 group; c vs. Zn 60 group; d vs. Cd group; e vs. Cd + Zn 30 group; ^†^ *p* < 0.01; ^‡^ *p* < 0.001. The values inside the bars express the percentage changes vs. the control group (↓, decrease; ↑, increase;) or Cd group (↙, decrease).

The concentrations of H_2_O_2_ and MPO in the brain tissue of rats maintained on the drinking water containing Zn alone were within the values noted in the control group (Figure 4).

The exposure to Cd increased the levels of H_2_O_2_ and MPO (by 60% and 37%, respectively), while the simultaneous administration of Zn at both concentrations entirely protected against the Cd-mediated increase in these parameters (Figure 4). MPO concentration in both groups co-administered with Ca and Zn was even lower than in the control rats (Figure 4). The differences between the level of Zn administration in the Cd exposed animals included the higher concentration of H_2_O_2_ and the lower that of MPO in the group supplemented with 60 mg Zn/L compared to the one received 30 mg Zn/L (Figure 4). The beneficial impact of Zn supplementation towards the brain H_2_O_2_ and MPO concentrations in the animals intoxicated with Cd was caused by this essential element itself (*F* = 10.95–73.27, *p* < 0.01–0.001) or its interaction with this xenobiotic (*F* = 36.89–75.84, *p* < 0.001) (Table 4).

### 3.4. The Impact of Cd and/or Zn on TAS and TOS and the Severity of Oxidative Stress in the Brain and Serum

**Figure 5:**
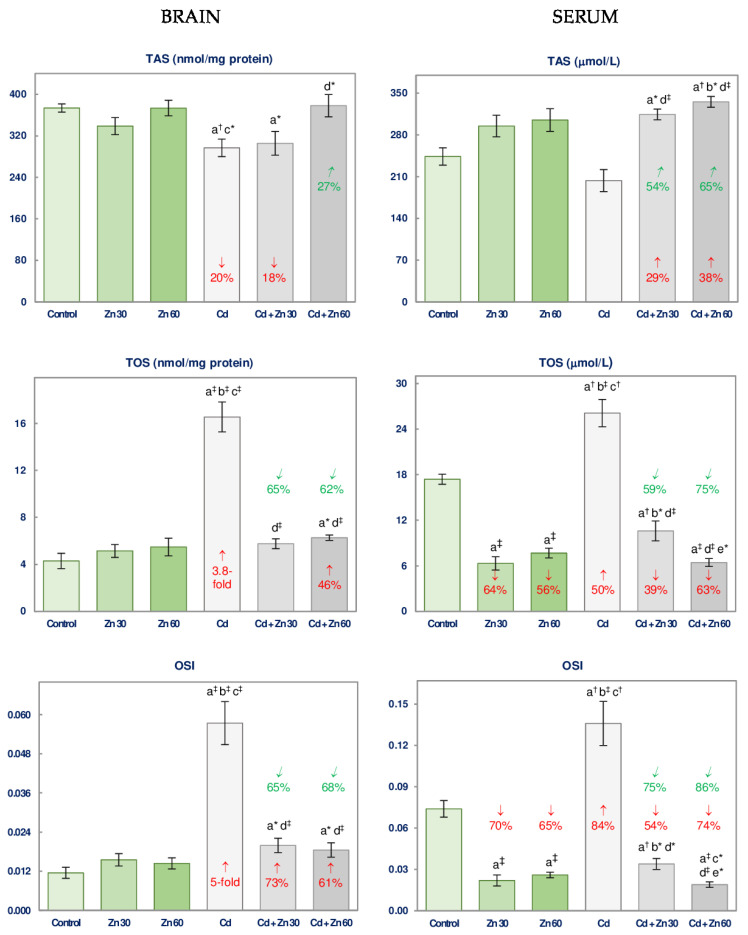
Total antioxidative status (TAS), total oxidative status (TOS), and oxidative stress index (OSI) in the brain tissue and serum of male rats after 6-month treatment with cadmium (5 mg Cd/L) and/or zinc (30 or 60 mg Zn/L) via drinking water. Data are shown as mean ± standard error (SE) for 8 animals. Statistically significant differences: a vs. Control group; b vs. Zn 30 group; c vs. Zn 60 group; d vs. Cd group; e vs. Cd + Zn 30 group; * *p* < 0.05; ^†^ *p* < 0.01; ^‡^ *p* < 0.001. The values inside the bars (or above them) express the percentage changes or folds of changes vs. the control group (↓, decrease; ↑, increase) or Cd group (↙, decrease; ↗, increase).

TAS and TOS and the value of OSI in the brain were not influenced by the administration of 30 or 60 mg Zn/L alone (Figure 5).

The administration of Cd decreased the brain TAS (by 20%) and increased TOS and OSI (by 3.8- and 5-fold, respectively) (Figure 5). The supplementation with 30 or 60 mg Zn/L under Cd treatment provided complete or partial protection against this xenobiotic influence on the investigated parameters, except for TAS in the Cd + Zn 30 group, which was unaffected by Zn (Figure 5). There were no differences in the investigated parameters in the brain depending on Zn dosage during the intoxication with Cd (Figure 5). The effect of the application of Zn to the rats received Cd on the values of TAS and TOS, as well as OSI in the brain was the main effect of Zn action (*F* = 6.334–39.45, *p* < 0.05–0.001) and its antagonistic interaction with this unnecessary element (*F* = 6.379–53.86, *p* < 0.05–0.001) (Table 5). The two-way analysis of variance confirmed the lack of impact of 30 mg Zn/L on the brain TAS in the animals exposed to Cd (Figure 5). Based on the analysis, the decrease in TAS in the animals co-administered with Cd and 30 mg Zn/L was the main effect of Cd action (*F* = 10.55, *p* < 0.01; Table 5).

The administration of Zn alone at both used concentrations caused a decline (by 56–70%) in the serum TOS and OSI (Figure 5), while the intoxication with Cd alone led to a significant increase in these variables (by 50% and 84%, respectively; Figure 5). Both Zn and Cd alone did not influence the serum TAS (Figure 5).

The supplementation with Zn under the treatment with Cd completely prevented the increase in the serum TOS and OSI, making them even lower (by 39–74%) compared to the control animals (Figure 5). The serum TAS in the Cd + Zn 30 and Cd + Zn 60 groups was higher (by 29–65%) compared to the control group and the group intoxicated with Cd alone (Figure 5). The administration of 60 mg Zn/L under the intoxication with Cd was more effective in decreasing the serum TOS and OSI than the lower dosage of this bioelement (Figure 5). The influence of the supplementation with Zn at the simultaneous treatment with Cd on the values of TAS, TOS, and OSI in the serum resulted from the independent Zn action (*F* = 27.32–198.6, *p* < 0.001) and/or this essential element interactive action with Cd (*F* = 5.042–22.74, *p* < 0.05–0.001) (Table 5).

### 3.5. The Impact of Cd and/or Zn on the Oxidative Injury of Lipids and Proteins in the Brain

**Figure 6:**
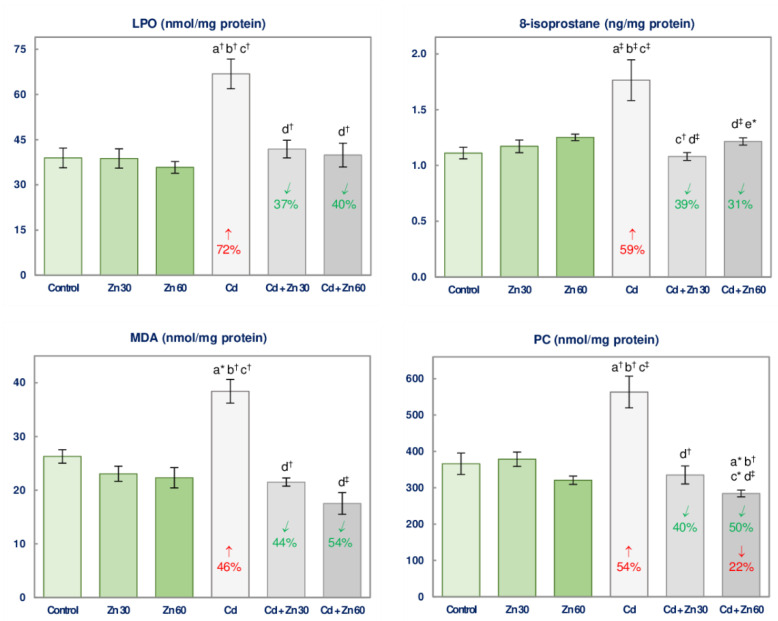
The concentrations of lipid peroxides (LPO), malondialdehyde (MDA), 8-isoprostane, and protein carbonyls (PC) in the brain tissue of male rats after 6-month treatment with cadmium (5 mg Cd/L) and/or zinc (30 or 60 mg Zn/L) via drinking water. Data are shown as mean ± standard error (SE) for 8 animals. Statistically significant differences: a vs. Control group; b vs. Zn 30 group; c vs. Zn 60 group; d vs. Cd group; e vs. Cd + Zn 30 group; * *p* < 0.05; ^†^ *p* < 0.01; ^‡^ *p* < 0.001. The values inside the bars express the percentage changes or folds of changes vs. the control group (↓, decrease; ↑, increase) or Cd group (↙, decrease).

The supplementation with Zn alone did not affect the biomarkers of lipids (LPO, MDA, and 8-isoprostane) and proteins (PC) oxidative damage in the brain (Figure 6).

The intoxication with Cd was a cause of an elevation (by 46–72%) in the concentrations of LPO, MDA, 8-isoprostane, and PC, while the supplementation with 30 or 60 mg Zn/L completely protected from these changes (Figure 6). The concentration of PC in the Cd + Zn 60 was even lower (by 22%) than in the control animals (Figure 6). The only difference in the values of these biomarkers dependent on the level of the intake of Zn at the treatment with Cd was the higher concentration of 8-isoprostane in the animals co-administered with 5 mg Cd/L and 60 mg Zn/L than in the Cd + Zn 30 group (Figure 6).

The beneficial outcomes of the supplementation with Zn on the concentrations of LPO, MDA, 8-isoprostane, and PC in the brain of the animals exposed to Cd resulted from the independent influence of this bioelement (*F* = 4.457–35.31, *p* < 0.05–0.001) and Cd-Zn interaction (*F* = 10.39–20.75, *p* < 0.01–0.001), which was antagonistic, except for the impossible to evaluate the character of the impact of Cd and 60 mg Zn/L on PC concentration (Table 6).

### 3.6. Cd and Zn Concentration in the Brain

**Figure 7:**
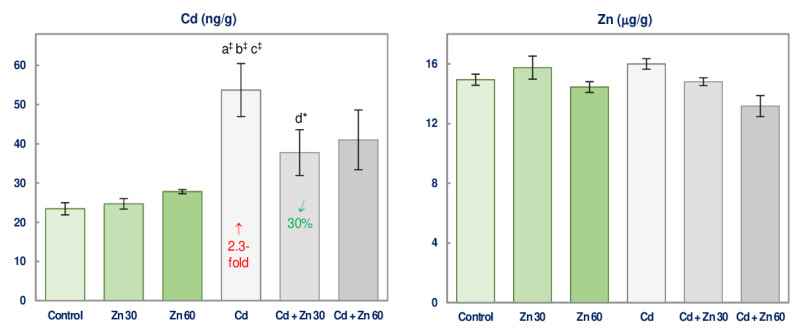
The concentrations of cadmium (Cd) and zinc (Zn) in the brain tissue of male rats after 6-month treatment with 5 mg Cd/L and/or 30 or 60 mg Zn/L via drinking water. Data are shown as mean ± standard error (SE) for 8 animals. Statistically significant differences: a vs. Control group; b vs. Zn 30 group; c vs. Zn 60 group; d vs. Cd group; * p < 0.05; ^‡^ *p* < 0.001. The values inside the bars express the percentage change or a fold of change vs. the control group (↑, increase) or Cd group (↙, decrease).

The maintenance of the rats on the drinking water containing only 30 or 60 mg Zn/L did not modify the concentration of Cd and Zn in the brain tissue (Figure 7).

In the animals intoxicated with 5 mg Cd/L for 6 months, the concentration of this toxic metal in the brain increased 2.3 times compared to the control ones, whereas Zn concentration was unaffected (Figure 7). The simultaneous administration of Cd and Zn (at both concentrations) did not influence this bioelement concentration in the brain but provided entire protection against Cd accumulation in this organ (Figure 7).

The ANOVA/MANOVA analysis showed a lack of the main effect of Zn and the effect of Cd-Zn interaction on the brain concentration of this xenobiotic (Table S2) despite the beneficial impact of this bioelement disclosed by the Kruskal-Wallis test (Figure 7). According to the two-way analysis of variance, Cd concentration in the Cd + Zn 30 and Cd + Zn 60 groups was determined mainly by the administration of this toxic element (*F* = 22.42 and *F* = 17.71, respectively, *p* < 0.001) (Table S2).

### 3.7. Correlations

The brain Cd concentration positively correlated with the main indices of the oxidative status such as TOS and OSI, and with H_2_O_2_ concentration (Table 7). Negative relationships occurred between this xenobiotic concentration and CAT activity, TSH concentration, and the GSH/GSSG ratio (Table 7). Apart from that, the concentration of GSSG in the brain positively correlated with Cd concentration (Table 7). The brain Cd concentration positively correlated with 8-isoprostane concentration but there were no relationships between this xenobiotic accumulation and the concentrations of LPO, MDA, and PC in this organ (Table 7).

Numerous negative correlations (*p* < 0.05–0.001) were noted between the markers of the antioxidative potential (SOD, GPx, CAT, GSH, GSH/GSSG) and the concentration of LPO (*r* = −0.327 to *r* = −0.461), MDA (*r* = −0.294 to *r* = −0.475), 8-isoprostane (*r* = −0.361 to *r* = −0.536), and PC (*r* = −0.311 to *r* = −0.470) (Table S3). The brain tissue activity of GR positively correlated with the concentrations of LPO, MDA, 8-isoprostane, and PC (*r* = 0.419–0.496, *p* < 0.05–0.001) (Table S3). The values of all markers of oxidative damage to lipids (LPO, MDA and 8-isoprostane) and proteins (PC) positively correlated (*r* = 0.312 to *r* = 0.707, *p* < 0.05–0.001) with the evaluated indices of oxidative status except for a lack of relationship between LPO and TOS, as well as MDA and H_2_O_2_ (Table S3). A tendency to negative dependence between LPO concentration and TSH concentration (*r* = −0.245, *p* = 0.05) or TAS value (*r* = −0.280, *p* = 0.05) was noted (Table S3). Positive correlations were noted between the concentrations of each two of the analyzed biomarkers of lipid peroxidation (*r* = 0.285 to *r* = 0.516, *p* < 0.05−0.001) (Table S3). Moreover, the concentration of PC positively correlated with the concentration of LPO (*r* = 0.307, *p* < 0.05) and MDA (*r* = 0.501, *p* < 0.001) but there was no dependence between the brain concentrations of PC and 8-isoprostane (Table S3).

There were no correlations between the values of TAS (*r* = 0.130, *p* > 0.05), TOS (*r* = 0.236, *p* > 0.05), and OSI (*r* = 0.170, *p* > 0.05) in the brain and serum.

## 4. Discussion

The present research indicates the protective effect of Zn against disruption of the oxidative/antioxidative balance resulting in the occurrence of the state of oxidative stress and oxidative injury of the basic cellular macromolecules in the brain under moderate repeated exposure to Cd. It is the first report not only on the beneficial impact of enhanced intake of Zn but also on the pro-oxidative action of Cd in the nervous system revealed in the animal model well reflecting human intoxication with this xenobiotic occurring in some industrialized countries [23,24,25,26,27,28,30]. Cd is a well-known neurotoxic agent [29,30,31,38] and the possible involvement of this xenobiotic in the pathogenesis of neurodegenerative diseases nowadays attracts the interest of scientists [29,30,31,32,33]. However, the evidence for Cd neurotoxicity comes mainly from experimental studies conducted in animal models [18,22,31,34,35,36,37,38,39,40,41,42,43,44,45,46] or brain endothelial cells [47,48], and the precise mechanism of its action on the nervous system is still far from being fully explained. Moreover, the risk of damage to the nervous tissue due to low and moderate chronic exposure is unknown.

This study shows that moderate treatment with Cd may disturb the balance between pro- and antioxidants in the nervous system by inhibiting the activities of enzymatic antioxidants (SOD, GPx, and CAT) and decreasing the concentrations of the non-enzymatic ones (GSH and TSH), as well as increasing pro-oxidants concentrations (H_2_O_2_ and MPO). An important finding is revealing that this effect occurs at the very low accumulation of this element in the brain tissue (0.0537 ± 0.0067 μg/g). Due to the function of the blood-brain barrier and the low induction of MT biosynthesis in the nervous tissue, the penetration of Cd into the brain and its accumulation in this organ are smaller compared to other organs, mainly the liver and kidneys [10,11,12,18,34,56] (Table S1); however, this xenobiotic may pass the barrier [47,48], as it was confirmed by our study, and cause serious disorders in the brain tissue even at its low concentration. The decrease in TAS and increase in TOS and OSI as a result of the exposure to Cd indicate that, despite the increased activity of GR, the antioxidative system of the brain tissue was not efficient enough to maintain the balance between antioxidants and pro-oxidants. The increase in the activity of GR could be a defense mechanism of the cells, as well as the effect of the increase in the concentration of GSSG being the substrate for GR [39,57]. The Cd-induced drop in the activities of antioxidative enzymes might be, first of all, the result of interactions between this xenobiotic and bioelements incorporated in the active centers of these enzymes: Zn, manganese, and Cu in SOD, selenium in GPx, and Fe in CAT [2,36,58].

Although the brain concentration of PSH was unaffected due to the exposure to Cd, the decreased concentrations of TSH and GSH show that the weakening of the antioxidative defense resulted also from insufficient non-enzymatic protection. The unchanged concentration of PSH at the simultaneously decreased concentrations of TSH and GSH may suggest that proteins are less susceptible to modifications resulting in a decrease in the content of thiol groups (-SH groups) than other -SH-containing cellular molecules, including GSH. Under normal conditions, PSH constitute only a small fraction of the total -SH groups present in the brain (in control animals the concentration of PSH was 71 times lower than that of TSH) and thus the lack of change in the concentration of PSH did not mask the impact of Cd on the level of non-protein -SH groups, that, together with the protein-bound -SH groups, were determined as TSH. The Cd-induced decline in the concentration of TSH was partly due to the decrease in the concentration of GSH, which serves as a first line of non-enzymatic defense in ROS removal.

The Cd-caused increase in the concentration of H_2_O_2_ might be the result of its increased production and ineffective detoxification by the weakened antioxidative system, especially the decreased activity of CAT. The disturbances in the oxidative/antioxidative balance in the brain led to the lowering in TAS and raise in TOS and development of oxidative stress reflected in the enhanced value of OSI. As a consequence, oxidative changes in lipids and proteins were noted in the brain of the rats intoxicated with Cd. The decrease in the brain TAS in the Cd group at the simultaneous lack of a change of the value of this parameter in the serum shows that the brain may be especially susceptible to Cd-induced weakening of the antioxidative abilities. The value of TAS measured in the serum reflects the antioxidative status of the whole body and may be unaffected even if the oxidative/antioxidative balance is disturbed in some organs, as it was noted in this study in the case of the brain.

The elevated levels of LPO, MDA, and 8-isoprostanes noted in the brain tissue in the 5 mg Cd/L group indicate increased lipid peroxidation [11,15,38,39,44,49,50], whereas the elevated concentration of PC reflects oxidative modifications of proteins [39,49,53] in the nervous tissue. The damage to these essential cellular macromolecules may lead to very negative outcomes to the morphological structure and function of neurons [40,58]. Lipid peroxidation is the leading mechanism of Cd toxicity including its neurodegenerative action [20,21,33,37,40,47,48,49,59]. Oxidative modifications of lipids may cause disturbances in the fluidity of membranes, affect the membrane-bound enzymes (including Na^+^/K^+^-ATPase), and intracellular homeostasis of Ca [41,42]. Damage to proteins may lead to disturbances in the functioning of many enzymes and neurotransmitters [31,43]. The exposure to 5 mg Cd/L-induced oxidative modifications of proteins in the brain might also alter the function of antioxidative enzymes. Thus, the findings warrant the conclusion that even a slight accumulation of Cd in the brain may be dangerous for the nervous tissue and this is an important outcome of the present investigation. Oxidative stress, an enhanced lipid peroxidation, and decreased concentration of thiol-rich compounds in the brain have been reported in various experimental models of exposure to Cd [22,44,45]; however, the present study is the first that evaluated and revealed these effects in an in vivo model of actual lifetime human exposure to this toxic heavy metal and included a complex evaluation of the oxidative/reductive status of the nervous tissue. As the present experiment was carried out in an animal model of human intoxication with Cd, the findings allow the conclusion that moderate exposure to this toxic heavy metal may create a risk of damage to the brain tissue in humans.

The results of the present study taken together with our previous findings from investigations conducted in these animals [10,11,12,13,14,15] seem to suggest that the brain tissue may be more resistant to the unfavorable impact of this xenobiotic on the concentration of Zn than other organs and tissues. The 6-month administration of the drinking water containing 5 mg Cd/L, which did not influence the brain concentration of this essential element, increased its concentration in the main organs of Cd storage in the body such as kidneys and liver, while decreased that in the femur and serum [10,11,12]. Moreover, we have reported that the feeding with the 1 and 5 mg Cd/kg diet for 3–24 months, that modified the concentration of Zn in the kidney, liver, spleen, and bone tissue had no impact on its concentration in the brain [60]. Some other authors have also reported an unhanged concentration of this bioelement in the brain of rats repeatedly intoxicated with Cd [34,46]. A lack of change in the brain concentration of Zn was noted also in the case of acute treatment with this xenobiotic [45]. Mimouna et al. [18] reported a decline in the brain Zn concentration in pups of female rats intoxicated with Cd during gestation and lactation periods; however, they administered this toxic metal at a higher concentration than in the present study (50 mg Cd/L). Saleh et al. [44] noted a decrease in the brain concentration of this essential element as a result of oral treatment with 3 mg CdCl_2_/kg b.w. for 30 consecutive days.

Taking into account that the excess of Zn in the organism may also be dangerous [1,3,4,5,6], an important result of this study is revealing that the elevation of this bioelement intake by 79% and 151% in the animals received 30 and 60 mg Zn/L, respectively, does not pose a danger of disturbance in the oxidative/reductive balance and does not lead to retention of his essential element in the brain. Although the administration of 30 mg Zn/L caused a decrease in the concentrations of TSH and GSH and the GSH/GSSG ratio, and the higher Zn consumption (60 mg/L) decreased TSH concentration, the elevated intake of this essential element had no influence on the value of TAS and, as a result, did not lead to the development of oxidative stress in the brain tissue.

However, the most important outcome of the current investigation is showing that the enhanced Zn intake prevents the Cd-induced oxidative/antioxidative imbalance and oxidative modifications of lipids and proteins in the brain. The beneficial influence of this bioelement against the pro-oxidative impact of Cd was also reflected in its ability to counteract oxidative stress in the serum. Generally, both dosages of Zn were equally effective in combating the pro-oxidative Cd action; nevertheless, the effect of the higher one on the antioxidative ability (SOD, GPx, and GR) during the exposure to Cd seems to be more significant than that of the lower one. Moreover, it should be emphasized that the administration of Zn at the concentration of 30 and 60 mg/L provided complete protection from Cd-induced damage to lipids, as well as proteins in the brain. Taking into consideration that oxidative stress may lead to morphological changes of the neural cells and development of neurodegeneration [39,40,47,48,50,59], based on the findings it can be suspected that administration of Zn could also protect from structural changes in the brain; however, this issue requires further investigation.

Results of some other authors also suggested that Zn may protect from neurotoxic action of Cd by alleviation of lipid peroxidation [22]. Bernotiene et al. [22] revealed that 14-day i.p. administration of Zn (24 mmol Zn/kg b.w.) prevented lipid peroxidation (evaluated based on MDA concentration) in the brain of mice sub-acutely treated with Cd (14 mmol Cd/kg b.w., i.p.). However, Braga et al. [34] reported no protective impact of this bioelement (2 mg Zn/kg, i.p.) against enhanced lipid oxidation (reflected in an increased level of thiobarbituric acid-reactive substances) in the brain of rats treated with neurotoxic Cd doses such as 0.25 and 1 mg Cd/kg (i.p.) for 10 days.

The protective effect of the supplementation with 30 and 60 mg Zn/L on the oxidative/reductive balance in the brain and serum, noted in the animals treated with 5 mg Cd/L, resulted from the independent impact of this essential element, as well as its interactive action with this toxic metal. The independent action of Zn might be caused by its high antioxidative potential. The antioxidative properties of Zn are well known and widely described [2,3,17,21,22,37]. The findings of this study confirmed that the enhancement of the daily Zn intake by 79% and 151%, alone and under moderate treatment with Cd, has antioxidative potential. First of all, Zn is an essential component for the proper activity of SOD playing a key role in the detoxification of O_2_^−^ in the cells [2,3,17]. Secondly, it may influence cellular signal transduction and up-regulate the transcription factor-nuclear factor erythroid 2-related factor 2 (Nrf2), that regulate the expression of genes encoding antioxidants, including SOD and GSH [2,3,17,20]. The antioxidative properties of Zn may also be connected with the induction of MT synthesis [2,3,17,22,44]. However, the data on the effect of simultaneous administration of Zn and Cd on the brain homeostasis of this protein are sparse and need further evaluation. Besides the major function of MT is Zn storage, this molecule is also a potent radical scavenger and cytoprotective agent [2,3,17,22,44]. Moreover, MT possesses Cd chelating properties and by this action protects molecular targets from deleterious effects of this xenobiotic [17]. It has been shown that Cd downregulated the expression of the *MT3* messenger ribonucleic acid (mRNA) [44] and decreased MT content in the brain [22] and the reduction in the concentration of MT in this organ was associated with increased lipid peroxidation [24,32,44]. On the other hand, Braga et al. [34] have revealed no impact of Zn towards Cd-dependent reduction in the brain content of MT and suggested that the mechanism of protection provided by this element against Cd toxicity is not related to MT levels [34]. Moreover, Zn can reduce the toxic action of transition metals such as Fe and Cu and in this way prevent the formation of highly reactive hydroxyl radical [2,3]. Zn is also a very important element for the proper function of mitochondria [3]. Homeostasis of these organelles, being the main sources of ROS, is especially important to maintain the oxidative/reductive balance of the cell [3]. It is as well value to mention the anti-inflammatory properties of Zn [2,20]. Taking into account that inflammation may evoke the formation of ROS and free radicals, this ability of Zn may also contribute to its antioxidative effect [2,20].

The interactive action of Zn and Cd resulted from a direct competition of this bioelement with this toxic metal [10,20]. It is established that cadmium ions (Cd^2+^), due to the chemical similarity to Zn ions (Zn^2+^), may use the same transporter systems, including specific transporters for Zn^2+^ such as ZP8 and ZIP14, divalent metal transporter 1 (DMT1), and Ca channels, to enter the cells [17,36]. It has been shown that Zn^2+^ inhibits the cellular uptake of Cd^2+^ [17,20] and in our previous investigation conducted in the same experimental model, we have revealed that this bioelement reduced the blood concentration of Cd and this xenobiotic accumulation in the body, including the main organs of its storage (Table S1) [11,12]. The antagonistic interactive effect of Zn and Cd towards the investigated parameters describing the nervous tissue oxidative/reductive status may be explained by the ability of this bioelement to combat the pro-oxidative effect of Cd by lowering this unnecessary element accumulation in the brain. The fact that the ANOVA/MANOVA analysis did not reveal either an independent influence of Zn or this bioelement interactive impact with Cd on this xenobiotic concentration in the brain tissue might result from the noted in the present study its low accumulation in the brain. However, the finding that the brain tissue concentration of Cd in the animals co-administered with this toxic element and Zn reached the values noted in the control animals (while in the Cd group the concentration was 2.3 times higher compared to the control ones) clearly show that Zn administration protects against Cd accumulation in the nervous tissue. Taking into account that at the same daily intake of Cd in the Cd group, Cd + Zn 30 group, and Cd + Zn 60 group, the body burden of this xenobiotic in the rats co-administered with both elements was lower than in the animals treated with Cd alone [10,11,12] (Figure 7, Table S1) and that the concentration of Cd in the brain tissue in the group supplemented with Zn under the exposure to Cd did not differ compared to the control group, it may be concluded that the protective effect of Zn against the impact of Cd on the oxidative/antioxidative status of the nervous tissue might be importantly determined by lower gastrointestinal absorption of Cd and thus the lower amount of this toxic element available to penetrate into the brain.

This paper presents a part of the findings of our wide designed research on the protective potential of Zn at the condition of chronic exposure to Cd. The results of the current study, as well as our earlier outcomes showing the beneficial influence of Zn at the condition of intoxication with Cd [10,11,12,13,14,15], make it justifiable to assume that this element may protect from Cd-mediated damage to many organs and systems. However, because both deficiency and excess of Zn may be dangerous to health [1,3,4,5,6,7], it is important to find the specific range of this bioelement administration that will protect from the unfavorable Cd action and will not pose any danger of harmful effects of its over-supplementation. The results of this investigation, as well as our previous findings [10,11,12,13,14,15], indicate that, generally, both evaluated by us levels of Zn supplementation (30 and 60 mg/L) were effective in combating Cd toxicity; however, the lower one better protected from liver injury [12,14]. This may suggest that there is no need to enhance the intake of Zn by 151% to prevent the toxic effects of Cd and a 79% increase in its administration is definitely enough to do it.

Taking into account the growing prevalence of mental illnesses, increasing life expectancy, growing exposure to Cd, its long half-life (up to 30 years), and lifelong accumulation in the human organism, the neurotoxic action of this xenobiotic is a problem that cannot be neglected [23,32,33,61]. Therefore, searching for effective methods to prevent or diminish its toxic action is especially needed. The findings presented in this paper, supported by our earlier results and investigations of other authors [10,11,12,13,14,15,16,17,18,19,22,36,37], show that Zn is the promising neuroprotective factor in the case of Cd toxicity. Moreover, it should be mentioned that Zn was also noted to protect the brain from the damaging impact of other xenobiotics, including aluminum [62], lithium [63], and lead [45,64].

We are aware of some imperfections of our investigation. Its major limitation is that the experimental model consisted of males so the outcomes refer to males only. However, taking into account that females are reported to be more susceptible to Cd toxicity than males, due to low Fe stores and higher gastrointestinal absorption of this toxic metal [23], we could expect that Cd may lead to more severe destroying the oxidative/reductive balance in the female brain. Therefore, the efficacy of the Zn-provided protection may also differ, but it should occur. The next limitation of the study is that our findings on the protective role of Zn may do not apply to other than the oral route of both elements’ intake. However, taking into account that for the general population the main source of exposure to Cd is diet, it was very important to perform the study in the model of oral intoxication. Moreover, the findings of the ANOVA/MANOVA analysis showing that the beneficial impact of Zn on the oxidative/reductive status of the brain exposed to Cd resulted not only from interactive action of Cd and Zn, but also from the independent impact of this essential element may suggest that the protection is also possible at other routes of exposure, but it needs further studies.

## 5. Conclusions

This research indicates, for the first time, that Cd may induce oxidative stress at its low concentration in the brain tissue and that Zn protects against Cd-mediated development of oxidative stress and its consequences such as oxidative injury of lipids and proteins in the brain in the rat model of current environmental human exposure to this xenobiotic. A significant finding is also revealing that enhanced intake of Zn does not pose a danger of disturbance in the oxidative/reductive balance of the nervous tissue and this essential element retention. The outcomes of this study are another confirmation of a persisting need to investigate the effective means to prevent Cd-mediated damage to the organism, including the brain. Taking into account our findings on the effectiveness of Zn administration in combating harmful action of Cd, it seems justifiable to indicate this essential micronutrient as a possible promising factor to prevent the toxicity of this heavy metal at oral exposure, including its neurotoxic action.

## Figures and Tables

**Table 1:** Zinc (Zn) and cadmium (Cd) daily intakes via drinking water in particular experimental groups ^1,2^.

Experimental Group	Intake of Zn Mean/Range (mg/kg b.w./24 h)	Intake of Cd Mean/Range (mg/kg b.w./24 h)
Control	0	0
Zn 30	2.210 ± 0.083/1.260–3.670 ^3^	0
Zn 60	4.460 ± 0.649/2.400–6.410	0
Cd	0	0.436 ± 0.019/0.222–0.731
Cd + Zn 30	2.230 ± 0.085/1.330–3.570	0.423 ± 0.019/0.243–0.745
Cd + Zn 60	4.490 ± 0.166/2.410–6.670	0.432 ± 0.020/0.260–0.740

^1^ The rats received Zn and Cd in drinking water containing 30 or 60 mg Zn/L and/or 5 mg Cd/L. ^2^ Because of low concentrations of both metals in redistilled water used as a source of drinking water in the control group and to the preparation of the drinking fluids with Zn or/and Cd, the intake of these elements in the control animals was recognized to be 0 [10,11]. There were no differences (Kruskal-Wallis test) in the daily intake of Zn or Cd regardless of their administration (separately or jointly). ^3^ Data represent the mean ± standard error (SE) Zn or Cd intake per day throughout the 6-month study, as well as the range of particular elements intakes during the experiment for 8 animals in each group. The intakes of Zn and Cd were calculated based on the daily consumption of drinking water and particular metals concentration in this fluid.

**Table 2:** Interactive and main effects of cadmium (Cd) and zinc (Zn) on the activities of superoxide dismutase (SOD), glutathione peroxidase (GPx), glutathione reductase (GR), and catalase (CAT), in the brain tissue of male rats ^1,2^.

Parameter	5 mg Cd/L + 30 mg Zn/L				5 mg Cd/L + 60 mg Zn/L			
	Main Effect of Cd	Main Effect of Zn	Interactive Effect of Cd + Zn	Cd + Zn Effect vs. Cd Effect + Zn Effect Character of Cd-Zn Interaction	Main Effect of Cd	Main Effect of Zn	Interactive Effect of Cd + Zn	Cd + Zn Effect vs. Cd Effect + Zn Effect Character of Cd-Zn Interaction
**SOD**	17.47 ^‡^	NS	NS	No interaction	NS	19.68 ^‡^	10.17 ^†^	+24 ^3^ vs. −29 + 0 +24 vs. −29 ^4^
**GPx**	8.048 ^†^	NS	NS	No interaction	NS	8.596 ^†^	5.412 *	0 vs. −30 + 0 0 vs. −30 Antagonism
**GR**	27.33 ^‡^	35.80 ^‡^	16.81 ^‡^	0 vs. +90 + 0 0 vs. +90 Antagonism	4.274 *	30.93 ^‡^	43.30 ^‡^	−36 vs. +90 + 0 −36 vs. +90 ^4^
**CAT**	13.99 ^‡^	7.307 *	5.000 *	0 vs. −36 + 0 0 vs. −36 Antagonism	19.49 ^‡^	9.254 ^†^	NS	No interaction

^1^ The outcomes of the ANOVA/MANOVA statistical test are expressed as *F* values and the level of statistical significance (*p*). *F* values having *p* < 0.05 are recognized statistically significant (where * *p* < 0.05, ^†^ *p* < 0.01, ^‡^ *p* < 0.001). NS—a lack of statistically significant effect (*p* > 0.05). ^2^ To describe the possible character of the interaction between Cd and Zn, the effect of co-administration of the two elements was compared to the sum of the effects of their action in the case of separate administration (Cd + Zn 30 effect vs. Cd effect + Zn 30 effect and Cd + Zn 60 effect vs. Cd effect + Zn 60 effect). The effects of Cd, Zn 30, Zn 60, Cd + Zn 30, and Cd + Zn 60 are shown as percentage differences (−, decrease; +, increase) vs. the control. The interaction between Cd and Zn (Cd-Zn 30 or Cd-Zn 60) was considered as antagonistic (antagonism) when the effect of co-administration of Cd and Zn (expressed as a percentage difference vs. the control) was below the sum of the effects noted in the case of their separate administration. ^3^ The values express percentage differences. ^4^ The description of the character of the interaction between Cd and Zn was impossible.

**Table 3:** Interactive and main effects of cadmium (Cd) and zinc (Zn) on the concentrations of total thiol groups (TSH), reduced glutathione (GSH), and oxidized glutathione (GSSG), as well as the GSH/GSSG ratio in the brain tissue of male rats ^1,2^.

Parameter	5 mg Cd/L + 30 mg Zn/L				5 mg Cd/L + 60 mg Zn/L			
	Main Effect of Cd	Main Effect of Zn	Interactive Effect of Cd + Zn	Cd + Zn Effect vs. Cd Effect + Zn Effect Character of Cd-Zn Interaction	Main Effect of Cd	Main Effect of Zn	Interactive Effect of Cd + Zn	Cd + Zn Effect vs. Cd Effect + Zn Effect Character of Cd-Zn Interaction
**TSH**	69.10 ^‡^	31.31 ^‡^	49.57 ^‡^	−48 ^3^ vs. −53 + (−44) −48 vs. −97 Antagonism	15.94 ^‡^	5.051 *	49.70 ^‡^	−30 vs. −53 + (−45) −30 vs. −98 Antagonism
**GSH**	4.443 *	4.468 *	21.22 ^‡^	−22 vs. −35 + (−35) −22 vs. −70 Antagonism	27.35 ^‡^	NS	NS	No interaction
**GSSG**	NS	124.4 ^‡^	62.07 ^‡^	−37 vs. +31 + (−12) −37 vs. +19 ^4^	7.579 *	24.69 ^‡^	10.20 ^†^	0 vs. +31 + 0 0 vs. +31 Antagonism
**GSH/GSSG**	NS	21.98 ^‡^	102.3 ^‡^	+24 vs. −51 + (−27) +24 vs. −78 ^4^	29.43 ^‡^	8.425 ^†^	4.364 *	0 vs. −51 + 0 0 vs. −51 Antagonism

^1^ The outcomes of the ANOVA/MANOVA statistical test are expressed as *F* values and the level of statistical significance (*p*). *F* values having *p* < 0.05 are recognized statistically significant (where * *p* < 0.05, ^†^ *p* < 0.01, ^‡^ *p* < 0.001). NS—a lack of statistically significant effect (*p* > 0.05). ^2^ To describe the possible character of the interaction between Cd and Zn, the effect of co-administration of the two elements was compared to the sum of the effects of their action in the case of separate administration (Cd + Zn 30 effect vs. Cd effect + Zn 30 effect and Cd + Zn 60 effect vs. Cd effect + Zn 60 effect). The effects of Cd, Zn 30, Zn 60, Cd + Zn 30, and Cd + Zn 60 are shown as percentage differences (−, decrease; +, increase) vs. the control. The interaction between Cd and Zn (Cd-Zn 30 or Cd-Zn 60) was considered as antagonistic (antagonism) when the effect of co-administration of Cd and Zn (expressed as a percentage difference vs. the control) was below the sum of the effects noted in the case of their separate administration. ^3^ The values express percentage differences. ^4^ The description of the character of the interaction between Cd and Zn was impossible.

**Table 4:** Interactive and main effects of cadmium (Cd) and zinc (Zn) on the concentrations of hydrogen peroxide (H_2_O_2_) and myeloperoxidase (MPO) in the brain tissue of male rats ^1,2^.

Parameter	5 mg Cd/L + 30 mg Zn/L				5 mg Cd/L + 60 mg Zn/L			
	Main Effect of Cd	Main Effect of Zn	Interactive Effect of Cd + Zn	Cd + Zn Effect vs. Cd Effect + Zn Effect Character of Cd-Zn Interaction	Main Effect of Cd	Main Effect of Zn	Interactive Effect of Cd + Zn	Cd + Zn Effect vs. Cd Effect + Zn Effect Character of Cd-Zn Interaction
**H_2_O_2_**	29.81 ^‡^	20.42 ^‡^	63.25 ^‡^	0 vs. +60 ^3^ + 0 0 vs. +60 Antagonism	50.14 ^‡^	10.95 ^†^	36.89 ^‡^	0 vs. +60 + 0 0 vs. +60 Antagonism
**MPO**	19.59 ^‡^	71.50 ^‡^	51.54 ^‡^	−13 vs. +37 + 0 −13 vs. +37 ^4^	NS	73.27 ^‡^	75.84 ^‡^	−27 vs. +37 + 0 −27 vs. +37 ^4^

^1^ The outcomes of the ANOVA/MANOVA statistical test are expressed as *F* values and the level of statistical significance (*p*). *F* values having *p* < 0.05 are recognized statistically significant (where ^†^ *p* < 0.01, ^‡^ *p* < 0.001). NS—a lack of statistically significant effect (*p* > 0.05). ^2^ To describe the possible character of the interaction between Cd and Zn, the effect of co-administration of the two elements was compared to the sum of the effects of their action in the case of separate administration (Cd + Zn 30 effect vs. Cd effect + Zn 30 effect and Cd + Zn 60 effect vs. Cd effect + Zn 60 effect). The effects of Cd, Zn 30, Zn 60, Cd + Zn 30, and Cd + Zn 60 are shown as percentage differences (−, decrease; +, increase) vs. the control. The interaction between Cd and Zn (Cd-Zn 30 or Cd-Zn 60) was considered as antagonistic (antagonism) when the effect of co-administration of Cd and Zn (expressed as a percentage difference vs. the control) was below the sum of the effects noted in the case of their separate administration. ^3^ The values express percentage differences. ^4^ The description of the character of the interaction between Cd and Zn was impossible.

**Table 5:** Interactive and main effects of cadmium (Cd) and zinc (Zn) on total antioxidative status (TAS), total oxidative status (TOS), and oxidative stress index (OSI) in the brain tissue and serum of male rats ^1,2^.

Parameter	5 mg Cd/L + 30 mg Zn/L				5 mg Cd/L + 60 mg Zn/L			
	Main Effect of Cd	Main Effect of Zn	Interactive Effect of Cd + Zn	Cd + Zn Effect vs. Cd Effect + Zn Effect Character of Cd-Zn Interaction	Main Effect of Cd	Main Effect of Zn	Interactive Effect of Cd + Zn	Cd + Zn Effect vs. Cd Effect + Zn Effect Character of Cd-Zn Interaction
**BRAIN**								
**TAS**	10.55 ^†^	NS	NS	No interaction	5.021 *	6.334 *	6.379 *	0 vs. −20 ^3^ + 0 0 vs. −20 Antagonism
**TOS**	65.67 ^‡^	39.45 ^‡^	53.86 ^‡^	0 vs. +3.8-fold + 0 0 vs. +3.8-fold Antagonism	64.09 ^‡^	31.35 ^‡^	49.61 ^‡^	+46 vs. +3.8-fold + 0 +46 vs. 3.8-fold Antagonism
**OSI**	46.66 ^‡^	20.80 ^‡^	31.89 ^‡^	+73 vs. +5-fold + 0 +73 vs. +5-fold Antagonism	46.78 ^‡^	24.30 ^‡^	32.73 ^‡^	+61 vs. +5-fold + 0 +61 vs. +5-fold Antagonism
**SERUM**								
**TAS**	NS	27.32 ^‡^	NS	No interaction	NS	37.50 ^‡^	5.042 *	+38 vs. 0 + 0 +38 vs. 0 ^4^
**TOS**	27.30 ^‡^	114.9 ^‡^	NS	No interaction	12.88 ^†^	198.6 ^‡^	22.74 ^‡^	+63 vs. +50 + (−56) +63 vs. −6 ^4^
**OSI**	17.56 ^‡^	74.10 ^‡^	8.103 ^†^	−54 vs. +84 + (−70) −54 vs. +14 ^4^	10.77 ^†^	91.43 ^‡^	16.16 ^‡^	−74 vs. +84 + (−65) −74 vs. +19 ^4^

^1^ The outcomes of the ANOVA/MANOVA statistical test are expressed as *F* values and the level of statistical significance (*p*). *F* values having *p* < 0.05 are recognized statistically significant (where * *p* < 0.05, ^†^ *p* < 0.01, ^‡^ *p* < 0.001). NS—a lack of statistically significant effect (*p* > 0.05). ^2^ To describe the possible character of the interaction between Cd and Zn, the effect of co-administration of the two elements was compared to the sum of the effects of their action in the case of separate administration (Cd + Zn 30 effect vs. Cd effect + Zn 30 effect and Cd + Zn 60 effect vs. Cd effect + Zn 60 effect). The effects of Cd, Zn 30, Zn 60, Cd + Zn 30, and Cd + Zn 60 are shown as percentage differences (−, decrease; +, increase) vs. the control. The interaction between Cd and Zn (Cd-Zn 30 or Cd-Zn 60) was considered as antagonistic (antagonism) when the effect of co-administration of Cd and Zn (expressed as a percentage difference vs. the control) was below the sum of the effects noted in the case of their separate administration. ^3^ The values express percentage differences. ^4^ The description of the character of the interaction between Cd and Zn was impossible.

**Table 6:** Interactive and main effects of cadmium (Cd) and zinc (Zn) on the concentrations of lipid peroxides (LPO), malondialdehyde (MDA), 8-isoprostane, and protein carbonyls (PC) in the brain tissue of male rats ^1,2^.

Parameter	5 mg Cd/L + 30 mg Zn/L				5 mg Cd/L + 60 mg Zn/L			
	Main Effect of Cd	Main Effect of Zn	Interactive Effect of Cd + Zn	Cd + Zn Effect vs. Cd Effect + Zn Effect Character of Cd-Zn Interaction	Main Effect of Cd	Main Effect of Zn	Interactive Effect of Cd + Zn	Cd + Zn Effect vs. Cd Effect + Zn Effect Character of Cd-Zn Interaction
**LPO**	17.81 ^‡^	11.69 ^†^	11.37 ^†^	0 vs. +72 ^3^ + 0 0 vs. +72 Antagonism	18.47 ^‡^	16.56 ^‡^	10.39 ^†^	0 vs. +72 + 0 0 vs. +72 Antagonism
**MDA**	12.42 ^†^	45.01 ^‡^	20.75 ^‡^	0 vs. +46 + 0 0 vs. +46 Antagonism	3.824 ^#^	43.68 ^‡^	20.19 ^‡^	0 vs. +46 + 0 0 vs. +46 Antagonism
**8-isoprostane**	7.857 ^†^	9.644 ^†^	13.79 ^‡^	0 vs. +59 + 0 0 vs. +59 Antagonism	10.08 ^†^	4.457 *	16.65 ^†^	0 vs. +59 + 0 0 vs. +59 Antagonism
**PC**	6.302 *	12.36 ^†^	15.39 ^‡^	0 vs. +54 + 0 0 vs. +54 Antagonism	8.679 ^†^	35.31 ^‡^	18.31 ^‡^	−22 vs. +54 + 0 −22 vs. +54 ^4^

^1^ The outcomes of the ANOVA/MANOVA statistical test are expressed as *F* values and the level of statistical significance (*p*). *F* values having *p* < 0.05 are recognized statistically significant (where * *p* < 0.05, ^†^ *p* < 0.01, ^‡^ *p* < 0.001, ^#^ *p* = 0.06). ^2^ To describe the possible character of the interaction between Cd and Zn, the effect of co-administration of the two elements was compared to the sum of the effects of their action in the case of separate administration (Cd + Zn 30 effect vs. Cd effect + Zn 30 effect and Cd + Zn 60 effect vs. Cd effect + Zn 60 effect). The effects of Cd, Zn 30, Zn 60, Cd + Zn 30, and Cd + Zn 60 are shown as percentage differences (−, decrease; +, increase) vs. the control. The interaction between Cd and Zn (Cd-Zn 30 or Cd-Zn 60) was considered as antagonistic (antagonism) when the effect of co-administration of Cd and Zn (expressed as a percentage difference vs. the control) was below the sum of the effects noted in the case of their separate administration. ^3^ The values express percentage differences. ^4^ The description of the character of the interaction between Cd and Zn was impossible.

**Table 7:** Mutual dependencies between cadmium (Cd) concentration in the brain and markers of oxidative/antioxidative status of this organ ^1^.

Indices of Antioxidative Status		Indices of Oxidative Status		Markers of Oxidative Damage to the Cellular Macromolecules	
Parameter	*r ^p^*	Parameter	*r ^p^*	Parameter	*r ^p^*
SOD	NS	H_2_O_2_	0.344 *	LPO	NS
CAT	−0.407 ^†^	MPO	NS	MDA	NS
GPx	NS	TOS	0.525 ^‡^	8-isoprostane	0.365 *
GR	NS	OSI	0.388 ^†^	PC	NS
TSH	−0.364 *				
PSH	NS				
GSH	NS				
GSSG	0.356 *				
GSH/GSSG	−0.335 *				
TAS	NS				

^1^ Data are expressed as the values of correlation coefficient (*r*) and the level of statistical significance (*p*). The values of *r* with *p* < 0.05 were considered statistically significant (* *p* < 0.05, ^†^ *p* < 0.01, ^‡^ *p* < 0.001). NS—not statistically significant (*p* > 0.05). SOD, superoxide dismutase; CAT, catalase; GPx, glutathione peroxidase; GR, glutathione reductase; TSH, total thiol groups; PSH, protein thiol groups; GSH, reduced glutathione; GSSG, oxidized glutathione; TAS, total antioxidative status; H_2_O_2_, hydrogen peroxide; MPO, myeloperoxidase; TOS, total oxidative status; OSI, oxidative stress index; LPO, lipid peroxides; MDA, malondialdehyde; PC, protein carbonyls.

## Data Availability

The data presented in this study are available on request from the corresponding authors. The data are not publicly available.

## Supplementary Materials

The following are available online at https://www.mdpi.com/2072-6643/13/2/478/s1, Table S1: The effect of zinc (Zn) on the concentration of cadmium (Cd) in the liver and kidney of rats, Table S2: The main and interactive effects of cadmium (Cd) and zinc (Zn) on the concentration of Cd in the brain tissue of male rats. Table S3: Mutual dependencies between the indices of oxidative/antioxidative status of the brain tissue of male rats.

## Abbreviations

AAS	atomic absorption spectrophotometry
ANOVA/MANOVA	two-way analysis of variance
b.w.	body weight
Ca	calcium
CAT	catalase
Cd	cadmium
Cd^2+^	cadmium ion
CdCl_2_	cadmium chloride
Cu	copper
CV	coefficient of variation
ELISA	enzyme-linked immunosorbent assay
Fe	iron
GPx	glutathione peroxidase
GR	glutathione reductase
GSH	reduced glutathione
GSSG	oxidized glutathione
H_2_O_2_	hydrogen peroxide
i.p.	intraperitoneal, intraperitoneally
LPO	lipid peroxides
MDA	malondialdehyde
MPO	myeloperoxidase
MT	metallothionein
OSI	oxidative stress index
O_2_^−^	superoxide radicals
*p*	level of statistical significance
PC	protein carbonyls
PSH	protein thiol groups
*r*	correlation coefficient
ROS	reactive oxygen species
SE	standard error
SD	standard deviation
-SH	sulfhydryl group
SOD	superoxide dismutase
TAS	total antioxidative status
TOS	total oxidative status
TSH	total thiol groups
Zn	zinc
ZnCl_2_	zinc chloride
Zn^2+^	zinc ion
