# Supplementary material for: Enhanced Zinc Intake Protects against Oxidative Stress and Its Consequences in the Brain: A Study in an In Vivo Rat Model of Cadmium Exposure
Source: Nutrients. 2021 Jan 31;13(2):478. doi: 10.3390/nu13020478 (PMC7911633; doi:10.3390/nu13020478)
Supplement: Supplementary file 1 [file nutrients-13-00478-s001.pdf]

nutrients

Supplementary Materials

Table S1. The effect of zinc (Zn) on the concentration of cadmium (Cd) in the liver and kidney of rats [1,2,3].

Control (mean ± standard error)	Effect of Cd alone	5 mg Cd/L + 30 mg Zn/L		5 mg Cd/L + 60 mg Zn/L	
		Effect of Cd + Zn	Effect of Zn	Effect of Cd + Zn	Effect of Zn
**Liver**					
0.0369 ± 0.0049 μg/g	↑ 37-fold [a‡]	↑ 27-fold [a‡]	↙ 28% [d*]	↑ 25-fold [a‡]	↙ 33% [d*]
**Kidney**					
0.0694 ± 0.0040 μg/g	↑ 114-fold [a‡]	↑ 93-fold [a‡]	↙ 18% [d*]	↑ 89-fold [a‡]	↙ 22% [d*]

[1] The rats received 5 mg Cd/L of drinking water and 30 or 60 mg Zn/L of drinking water for 6 months. [2] Table presents changes in Cd concentration (Kruskal-Wallis post hoc test; * $p < 0.05$, ‡ $p < 0.001$): a vs. Control group (↑, fold of increase), d vs. Cd group (↙, percentage decrease). [3] Detailed data on the impact of Zn on the concentration of Cd in the organism of rats exposed to this xenobiotic have already been published [11,12].

Table S2. Interactive and main effects of cadmium (Cd) and zinc (Zn) on the concentration of Cd in the brain tissue of male rats [1].

5 mg Cd/L + 30 mg Zn/L				5 mg Cd/L + 60 mg Zn/L			
Main Effect of Cd	Main Effect of Zn	Interactive Effect of Cd + Zn	Cd + Zn Effect vs. Cd Effect + Zn Effect *Character of Cd – Zn Interaction*	Main Effect of Cd	Main Effect of Zn	Interactive Effect of Cd + Zn	Cd + Zn Effect vs. Cd Effect + Zn Effect *Character of Cd – Zn Interaction*
22.42 ‡	NS	NS	No interaction	17.71 ‡	NS	NS	No interaction

[1] The outcomes of the ANOVA/MANOVA statistical test are expressed as *F* values and the level of statistical significance (*p*). *F* values having $p < 0.05$ are recognized statistically significant (‡ $p < 0.001$). NS – not statistically significant ($p > 0.05$).

Table S3. Mutual dependencies between the indices of oxidative/antioxidative status of the brain tissue of male rats [1].

Parameter	SOD	CAT	GPx	GR	TSH	PSH	GSH	GSSG	GSH/GSSG	TAS	H_2O_2	MPO	TOS	OSI	LPO	MDA	8-Isoprostane
**SOD**	-																
**CAT**	0.388 †	-															
**GPx**	0.414 †	0.294 *	-														
**GR**	–0.448 †	NS	–0.308 *	-													
**TSH**	NS	NS	NS	–0.333 *	-												
**PSH**	NS	NS	NS	NS	NS	-											
**GSH**	NS	NS	NS	NS	NS	NS	-										
**GSSG**	NS	NS	NS	0.478 ‡	NS	NS	NS	-									
**GSH/GSSG**	NS	NS	NS	NS	NS	NS	0.608 ‡	–0.658 ‡	-								
**TAS**	0.472 ‡	NS	NS	NS	0.429 †	NS	0.430 †	NS	NS	-							
**H_2O_2**	NS	–0.371 †	NS	0.391 †	–0.315 *	NS	–0.392 †	0.429 †	–0.618 ‡	NS	-						
**MPO**	–0.398 †	NS	–0.411 †	0.651 ‡	–0.316 *	NS	NS	0.633 ‡	–0.369 †	NS	NS	-					
**TOS**	NS	–0.386 †	–0.428 ‡	0.348 *	–0.422 †	NS	–0.278 #	NS	–0.282 #	–0.288 *	0.422 †	0.336 *	-				
**OSI**	–0.360 *	–0.470 ‡	0.475 ‡	0.337 *	–0.517 ‡	NS	–0.447 †	NS	–0.278 #	–0.579 ‡	0.426 †	0.318 *	0.901 ‡	-			
**LPO**	–0.461 ‡	–0.392 †	–0.327 *	0.496 ‡	–0.245 #	NS	–0.357 †	NS	–0.386 †	–0.280 #	0.485 ‡	0.285 *	NS	0.352 †	-		
**MDA**	-0.294 *	NS	-0.385 †	0.494 ‡	NS	NS	NS	0.462 ‡	-0.475 ‡	NS	NS	0.707 ‡	0.368 *	0.328 *	0.430 †	-	
**8-Isoprostane**	NS	–0.366 †	NS	0.419 †	–0.361 *	NS	–0.367 †	0.403 †	–0.563 ‡	NS	0.595 ‡	0.452 †	0.537 ‡	0.502 ‡	0.285 *	0.516 ‡	-
**PC**	–0.470 ‡	NS	–0.375 †	0.476 ‡	–0.197 #	NS	–0.311 *	0.365 †	–0.391 †	–0.429 †	0.312 *	0.593 ‡	0.324 *	0.406 †	0.307 *	0.501 ‡	NS

[1] Data are expressed as *r* values and the level of statistical significance (*p*). The values of *r* with $p < 0.05$ were considered statistically significant (* $p < 0.05$, † $p < 0.01$, ‡ $p < 0.001$, # $p = 0.05$). NS – not statistically significant ($p > 0.05$). SOD, superoxide dismutase CAT, catalase; GPx, glutathione peroxidase; GR, glutathione reductase; TSH, total thiol groups; PSH, protein thiol groups; GSH, reduced glutathione; GSSG, oxidized glutathione; GSH/GSSG, the ratio of reduced glutathione to oxidized glutathione; TAS, total antioxidative status; H_2O_2, hydrogen peroxide; MPO, myeloperoxidase; TOS, total oxidative status; OSI, oxidative stress index; LPO, lipid peroxides; MDA, malondialdehyde; PC, protein carbonyls.
